# Effectiveness of fractionated rituximab in preventing tumor lysis syndrome in aggressive B‐cell lymphoma: Insights from real‐life clinical practice

**DOI:** 10.1002/cnr2.1983

**Published:** 2024-10-16

**Authors:** Jasmine Mohamad, Antonin Bouroumeau, Thomas A. McKee, Nicolas Mach, Kaveh Samii, Martine Chamuleau, Frank Stenner, Jerome Tamburini, Noémie Lang

**Affiliations:** ^1^ Translational Research Centre in Onco‐Hematology, Faculty of Medicine University of Geneva and Swiss Cancer Center Leman Geneva Switzerland; ^2^ Division of Clinical Pathology, Diagnostic Department Geneva University Hospital Geneva Switzerland; ^3^ Department of Patho‐Immunology Geneva University Medical Centre Geneva Switzerland; ^4^ Department of Oncology Hôpitaux Universitaires de Genève Geneva Switzerland; ^5^ Department of Hematology Hôpitaux Universitaires de Genève Geneva Switzerland; ^6^ Department Hematology Cancer Center Amsterdam Amsterdam Netherlands; ^7^ Department Oncology University Hospital Basel and SAKK Basel Switzerland

**Keywords:** aggressive B lymphoma, rituximab, tumor lysis syndrome

## Abstract

**Background:**

Tumor lysis syndrome (TLS) is a potentially life‐threatening condition resulting from the rapid destruction of malignant cells, leading to electrolyte imbalances and severe complications, such as acute kidney injury, arrhythmias, and seizures. TLS can be managed through hyperhydration, urate‐lowering treatments, and a steroid prophase strategy.

**Aims:**

This study aims to explore the impact of fractionated rituximab, an anti‐CD20 antibody, on the occurrence and severity of TLS during the initial cycle in patients with aggressive B‐cell non‐Hodgkin lymphoma (B‐NHL).

**Methods:**

Data was retrospectively collected from 94 of 186 patients.

**Results:**

Among the 94 patients included in the analysis, the median age was 70. Histologies were diffuse large B‐cell lymphoma (75%), Burkitt lymphoma (13%) and high‐grade B‐cell lymphoma (8%). The majority were at an advanced stage (93%) with a high IPI score (75%). Most patients received anthracycline‐containing regimens (72%) and prophylactic allopurinol (83%) and/or rasburicase (26%). Steroid prophase was administered to 82% of patients. The study identified one clinical TLS case and six laboratory TLS cases. Significant TLS factors included BL histology, elevated baseline LDH (⟩500 U/l), and rasburicase usage. Infusion reactions were rare (3%). Median progression‐free survival was 2.6 years, and 2‐year overall survival was 33%, irrespective of TLS occurrence.

**Conclusion:**

In this real‐life study, clinical TLS occurrence was low (1%). TLS appeared more frequent in BL but did not impact overall survival. Fractionated initial rituximab dosing in addition to preventive strategies is a feasible approach in preventing clinical TLS, warranting further prospective investigation.

## 1 INTRODUCTION

Tumor lysis syndrome (TLS) is a potentially life‐threatening condition caused by the rapid destruction of malignant cells leading to imbalances in various electrolytes, and posing risks such as acute kidney injury (AKI), arrhythmia, seizures, and potentially fatal outcomes if not managed appropriately.^1^ TLS primarily occurs in highly proliferative hematological malignancies such as acute leukemia and high‐grade non‐Hodgkin lymphomas (NHL), while its occurrence in solid tumors is less common.^2^ TLS is burdened with significant morbidity and mortality and may occur spontaneously and/or shortly after treatment initiation.^3^ Even though TLS can lead to fatal outcome, there have been limited studies focused on this condition.

TLS contributes to the formation of uric acid crystals and/or calcium phosphate salts, which deposit in renal tubules, resulting in acute obstruction and kidney failure.^4^ The precise occurrence of TLS remains uncertain, with historical reports indicating rates as high as 48%, including 6% clinically significant TLS in NHL.^5^ However, recent retrospective studies including various highly proliferative onco‐hematological malignancies reported TLS occurrence rates ranging from 5% to 16%.^3^, ^6^, ^7^ In approximately one‐quarter of cases, hemodialysis and intensive care unit interventions were required, while TLS‐related mortality ranged from 14% to 21%.^3^, ^7^, ^8^ Recognized clinical risk factors for TLS development include high tumor burden and proliferation index, increased sensitivity to chemotherapy, elevated white blood cell count, and preexisting renal impairment of the patient.^1^, ^9^ The estimated risk of TLS is 15% for Burkitt lymphoma (BL) and 6% for diffuse large B‐cell lymphoma (DLBCL).^1^

Early risk stratification based on tumor burden evaluation is required to efficiently prevent TLS. Intravenous hyperhydration along with urate‐lowering treatments such as xanthine oxydase inhibitors or uricolytic agents like the recombinant urate oxidase enzyme rasburicase have shown significant reduction in TLS occurrence.^8^, ^9^, ^10^, ^11^ Additionally, preemptive debulking approaches, involving steroids and/or low‐dose chemotherapy prophase, as well as ramp‐up dose schedule are used dependent on malignant condition and clinical assessment of tumor burden.^7^, ^11^, ^12^, ^13^

Rituximab, an anti‐CD20 monoclonal antibody is commonly used in combination with cytotoxic chemotherapy for the treatment of B‐cell malignancies.^14^, ^15^, ^16^, ^17^, ^18^ Early studies used fractionated rituximab to mitigate the risk of infusion reactions and TLS during the initial administration in low‐grade B‐cell malignancies.^19^, ^20^, ^21^, ^22^ Notably, TLS was reported as the second significant safety signal after rituximab administration in 1998.^19^ This approach is still utilized for bulky indolent lymphomas, including chronic lymphocytic leukemia, and is also extended to other CD20 antibodies, such as obinutuzumab.^19^, ^20^, ^21^, ^22^

In this study, we present a retrospective analysis of a single‐center experience on the use of fractionated rituximab as a debulking strategy in 94 patients with B‐cell malignancies at high‐risk for TLS over a 10‐year period.

## 2 METHODS

### 2.1 Patient cohort

A total of 186 charts of patients treated at the Hôpitaux Universitaires de Genève (HUG) with rituximab met the following inclusion criteria of newly diagnosed histologically proven aggressive B‐cell lymphoma and treated with at least two consecutive doses of fractionated rituximab 24 h apart during the first cycle of treatment. Patients were excluded if they had non‐B‐cell lymphoma histology, administration of a single dose of rituximab, required chronic dialysis, were under 18‐year‐old, and in the absence of written informed consent. The Ethics Committee of Geneva (CCER 2020‐01659) approved the study. Written informed consent was obtained in accordance with the declaration of Helsinki from alive patients (hospital general consent for clinical data use), and a waiver was applied for deceased patients when obtaining consent was not possible.

### 2.2 Data collection

Clinical data were extracted from electronic medical record system of the HUG, anonymized using a study‐specific patient identifier and collected in electronic certified clinical database management system software (REDCap™), an application compliant with the requirements of the Swiss clinical trial requirements. Deidentified data were analyzed and stored following Swiss legal requirements for data protection using ISO 9001/2008 standards. Health related personal data were accessible only by investigators. We employed the well‐established Cairo and Bishop criteria to define laboratory TLS (LTLS) or clinical TLS (CTLS),^18^ even though different algorithm management of TLS was recently proposed.^23^ LTLS involved 25% changes with baseline or upper normal value at two separate occasions (between 3 days before and 7 days after treatment onset) of uric acid, potassium, phosphorous, and calcium. CTLS was defined as serum creatinine increasing 1.5 times the upper normal values and/or seizure and/or cardiac arrhythmia.^18^ The Swiss Group for Clinical Cancer Research (SAKK) provided data from the Swiss BL cohort. Comparative clinical dataset from CALGB 50303 study were available through the National Cancer Institute (NCI) platform.

### 2.3 Endpoints

The primary endpoint of this study was to evaluate the occurrence of CTLS in patients with HGBCL treated with a fractionated rituximab schedule. Secondary endpoints were to report the occurrence of LTLS and other adverse events of interest (e.g., infusion reactions), explore clinical risk factors related with CTLS and report progression‐free survival and overall survival (OS) of the whole cohort, by TLS/no TLS and IPI subgroups.

### 2.4 Statistics

Binary and categorical variables were analyzed by calculating proportions, whereas continuous variables were assessed by calculating median and range. Missing data or irrelevant responses were excluded from the *p*‐value calculations. Fisher's exact test and *χ* ^2^ test were employed for categorical variables, whereas *t*‐tests were utilized for continuous variables. Significance was determined at a *p*‐value <.05. Survival analyses were presented using Kaplan–Meier curves. All calculations were performed using STATA software (v. 17.0).

## 3 RESULTS

**FIGURE 1:**
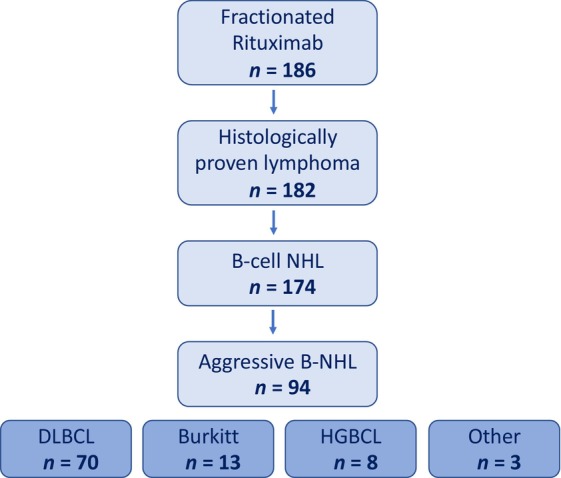
Flowchart of the study. DLBCL, diffuse large B cell lymphoma; HGBCL, high‐grade B cell lymphoma with MYC/BCL2/BCL6 rearrangements; NHL, non‐Hodgkin lymphoma.

We identified 186 patients who received rituximab between 2011 and 2020 at our institution, of whom, 94 met the study eligibility criteria. Patients with histologically unproven B‐NHL (*n* = 4), non‐B‐NHL (*n* = 8), indolent B‐NHL (*n* = 76), or high‐grade B‐NHL who only received a single dose of rituximab (*n* = 4), either because of reaction at infusion (*n* = 3) or cardiac arrhythmia (*n* = 1) were excluded from this analysis (Figure 1).

### 3.1 Patients' characteristics

Median age was 70 years, ranging from 31 to 95 years with a predominance of male (70%) (Tables 1 and S1). The histological subtypes were determined by the presence or absence of rearrangements in *MYC*, *BCL2*, or *BCL6*. The distribution of subtypes in the dataset is as follows: 75% were classified as: 75% DLBCL (*n* = 70), 14% BL (*n* = 13), 8% HGBCL (*n* = 8), and the remaining 3% were categorized in other subtypes. Most patients were diagnosed at advanced stage III/IV (*n* = 88, 93%) and were high risk based on the International Prognostic Index (IPI) score (*n* = 70, 75%). A bulk defined as tumor size larger than 10 cm along the longest axis was present in a quarter of patients.^24^, ^25^ Extranodal disease was present in most patients (*n* = 74, 80%), including central nervous system and bone marrow involvement in 13 and 23 patients, respectively. Despite most patients having a good performance status score of 0–1 (*n* = 59, 63%), a significant proportion displayed a Charlson comorbidity index (CCI) of more than 8 points (*n* = 23, 25%). It is noteworthy that 33% of patients (*n* = 31) exhibited abnormal glomerular filtration rate (GFR) values. The primary treatment approach utilized for most patients involved anthracycline‐containing regimens (*n* = 66, 72%) (Table S1).

**TABLE 1:** Characteristics of the patients (*n* = 94).

Age, median (min–max)	51 (18–75)
Gender, *n* (%)	
Male/female	14 (70)/6 (30)
Stage, *n* (%)	
Localized (I‐II)/advanced (III/IV)	2 (10)/18 (90)
B symptoms, *n* (%)	
Present	34 (36)
Missing	2 (2)
Extranodal disease, *n* (%)	
Present	74 (80)
Missing	2 (2)
Bulk, *n* (%)	
Present	25 (26)
Missing	1 (1)
IPI, *n* (%)	
0–2	22 (23)
3–5	70 (74)
Missing	2 (2)
Baseline LDH level (U/L), *n* (%)	
<500	48 (51)
500–1000	23 (25)
>1000	16 (17)
Missing	7 (8)
Baseline GFR (mL/min), *n* (%)	
>60	57 (61)
30–60	27 (29)
<30	4 (4)
Missing	6 (6)
CCI, *n* (%)	
2–4	22 (23)
5–7	43 (46)
>8	23 (25)
Missing	6 (6)
Aggressive B cell lymphoma subtype, *n* (%)	
DLBCL	70 (75)
BL	13 (14)
HGBCL	8 (9)
Other^a^	3 (3)

*Note*: extranodal disease including: bone marrow involvement *n* = 23 (25%) and CNS involvement *n* = 13 (14%).

Abbreviations: BL, Burkitt lymphoma; B‐NHL, B cell non‐Hodgkin lymphoma; CCI, Charlson comorbidity index; DLBCL, diffuse large B cell lymphoma; ECOG, Eastern Cooperative Oncology Group; GFR, glomerular filtration rate; HGBCL, high‐grade B cell lymphoma with MYC/BCL2/BCL6 rearrangements; IPI, International Prognostic Index; LDH, lactate dehydrogenase.

^a^ One follicular lymphoma grade 3B, one gray zone lymphoma and one post‐transplant lymphoproliferative disorder.

### 3.2 Occurrence of TLS, infusion reactions and preventive therapies

The median doses of rituximab were 224 mg (125–570 mg, 125 mg/m^2^), 224 mg (125–264 mg, 125 mg/m^2^) and 224 mg (125–574 mg, 125 mg/m^2^) on days 1, 2 and 3, respectively (Tables 2, 3, and S2). Allopurinol, given to most patients (77, 84%), was administered at a median dose of 300 mg/day (range: 50–300 mg/day). The average duration was found to be 13 days (range 0–107 days). Allopurinol initiation typically occurred 3 days before the first rituximab infusion (range Day −22 to Day +9). Rasburicase was given to 24 patients (26%) at a median dose of 15 mg/day (range: 7.5–22.5 mg/day). The average duration was found to be 4 days (range 0–15 days). Rasburicase initiation typically occurred 2 days before the first rituximab infusion (range Day −4 to Day +8). A large majority of patients received steroid prophase (*n* = 77, 82%). A single patient received just two doses of rituximab. Out of the entire cohort, seven patients (7.3%) experienced TLS. Among them, six cases (6%) were classified as LTLS, whereas one case (1%) was a CTLS. This CTLS case defined by Stage 3 AKI resolved following extensive fluid hydration. Among the LTLS cases, dialysis was required for one patient with BL due to severe metabolic alterations. Detailed characteristics of patients experiencing TLS are presented in Table 2. No significant associations were observed between TLS occurrence and factors such as elevated CCI scores (>8), elevated IPI scores (3–5), presence of bulky disease, use of allopurinol, use of steroid prophase, or the initial dose of rituximab infusion. However, TLS occurrence was significantly higher in cases with BL histology compared with those with DLBCL subtype, in patients with elevated LDH levels exceeding 500 U/L, in individuals with impaired GFR below 45 mL/min, and in patients who received rasburicase (Table 3). This last point indirectly reflects a high risk of TLS assessed by the clinician, considering factors such as a substantial tumor burden, renal insufficiency, or other elements placing the patient at risk of developing TLS. Infusion‐related reactions to rituximab were reported in three cases, all of which were classified as mild or moderate.

**TABLE 2:** Characteristics of patients experiencing TLS.

Age, median (range)	70 (56–93)
BL, *n* (%)	4 (57)
DLBCL, *n* (%)	2 (29)
Other^a^, *n* (%)	1 (14)
Stage IV, *n* (%)	6 (85)
Extranodal, *n* (%)	7 (100)
IPI 3–5, *n* (%)	6 (100)
Allopurinol, *n* (%)	7 (100)
Rasburicase, *n* (%)	4 (67)
Steroid prophase, *n* (%)	7 (100)

Abbreviations: BL, Burkitt lymphoma; DLBCL, diffuse large B cell lymphoma; TLS, tumor lysis syndrome.

^a^ One gray zone lymphoma.

**TABLE 3:** Clinical factors associated with TLS.

	TLS	no TLS	*p*‐value
IPI 3–5, *n* (%)	6 (86)	64 (74)	.57
Baseline LDH >500 U/L, *n* (%)	6 (16)	32 (8)	**.007**
Impaired baseline GFR (<45 mL/min)	3 (43)	9 (11)	**.043**
CCI score (>8)	1 (14)	22 (26)	.50
Bulk, *n* (%)	1 (14)	22 (27)	.67
BL subtype, *n* (%)	4 (57)	9 (10)	**.004**
Allopurinol, *n* (%)	6 (86)	71 (84)	.25
Rasburicase, *n* (%)	4 (57)	20 (24)	**.012**
Rituximab dose (mg), mean	220	225	.37
Steroid prophase use, *n* (%)	7 (100)	69 (80)	.342

Abbreviations: BL, Burkitt lymphoma; CCI, Charlson comorbidity index; GFR, glomerular filtration rate; IPI, international prognostic index; LDH, lactate dehydrogenase; TLS, tumor lysis syndrome.

### 3.3 Survival outcomes

**FIGURE 2:**
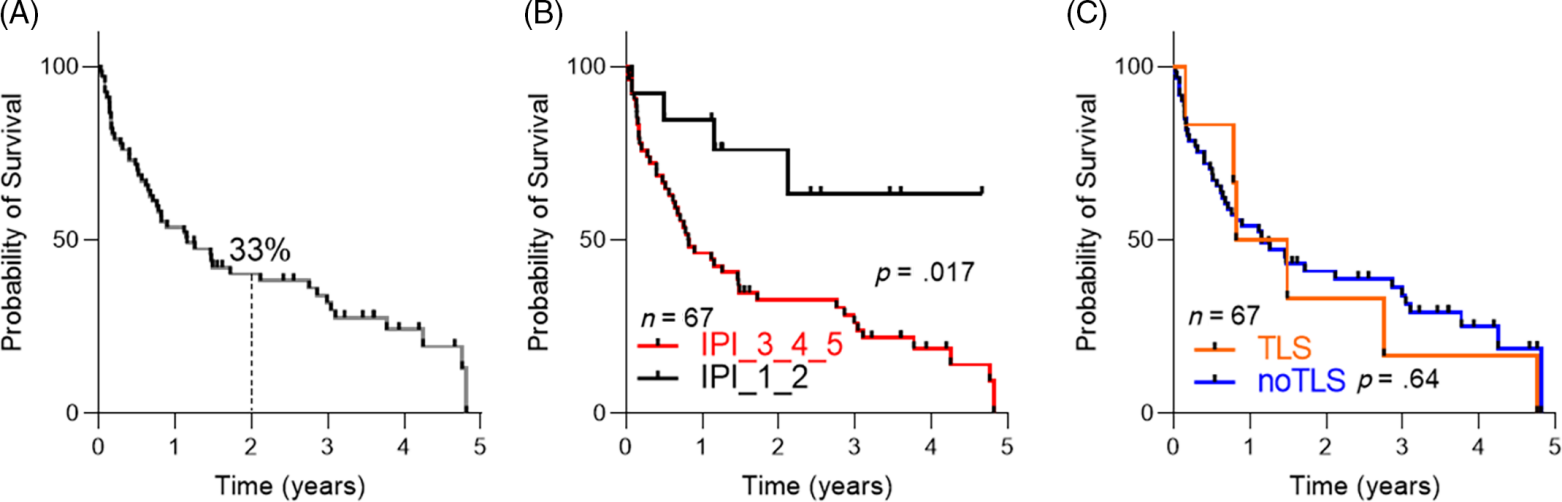
Overall survival (OS) in the real‐life cohort in 67 evaluable patients. (A) OS. The 2‐year OS probability is provided. (B) OS stratified per International Prognostic Index (IPI). IPI score was stratified as low (scores 1–2) or high (scores 3–5). (C) OS stratified per tumor lysis syndrome (TLS). *p*‐values are provided.

The 2‐year OS of the whole cohort was 33% (Figure 2A). When assessing the impact of clinical factors on survival, it was found that an elevated IPI score ranging from 3 to 5 was indicative of a poorer outcome (*p* = .017, Figure 2B). Despite the limited number of events (*n* = 7), there appears to be no significant association between the occurrence of TLS and altered survival outcomes (*p* = .64, Figure 2C).

### 3.4 Comparison of TLS occurrence between datasets of lymphoma patients

To assess the occurrence of TLS, we conducted a comparative analysis of our cohort with other datasets (Tables S3). First, we examined the Swiss BL dataset consisting of 20 cases^26^ (Table S3A). In our cohort, which included 13 cases of BL, no CTLS cases were observed, whereas four cases of LTLS were documented. In the Swiss BL dataset, CTLS was reported in 5% (1 out of 20 cases), and information regarding LTLS occurrence was not available. Additionally, we compared patients with DLBCL from our cohort (*n* = 78) with the CALGB 50303 (NCT0011820) dataset comprising 473 patients. Patients in CALGB 50303 (NCT0011820) did not receive steroids before immunochemotherapy. Unfortunately, data regarding allopurinol or rasburicase usage are unavailable. Within our cohort, one CTLS case (1.3%) and two LTLS cases were identified. In the CALGB 50303 dataset, CTLS occurred in 0.85% of patients (Table S3B), whereas no information regarding LTLS cases was reported.

## 4 DISCUSSION

In our retrospective study, we observe the occurrence of TLS among a real‐world cohort of high‐risk aggressive B‐cell lymphoma patients, as per the defined criteria.^1^ Accordingly, a fractionated rituximab approach was implemented as the initial strategy for TLS prevention in agreement with the guidelines of our institution. This approach was complemented by usual TLS prophylactic measures, including hyperhydration, and the use of allopurinol or rasburicase, based on the physician's evaluation of TLS risk. Additionally, it is worth noting that most patients received steroids (Table S2). The overall occurrence of TLS in our study was 7% including 1% CTLS and 6% LTLS, aligning with findings from other reports.^5^, ^6^, ^7^, ^23^, ^27^, ^28^, ^29^, ^30^, ^31^, ^32^ Notably, the occurrence of TLS had no significant impact on OS.

In our study population, the prevalence of TLS was higher among BL patients, constituting 57% of all TLS cases. Preliminary findings from the EudraCT 2013‐004394‐27 BL cohort (*n* = 89) indicated a TLS occurrence of 2.3% (2/89).^26^ Conversely, the LMBA02 trial, which included only 14% of patients aged 60 or above, did not report any cases of TLS resulting in renal failure higher than grade 2.^33^ In a cohort comprising 113 patients with BL who underwent treatment with the DA‐EPOCH‐R regimen, the occurrence of TLS was documented at a rate of 5%.^28^ This observation is consistent with prior studies where BL patients were included as subsets within diverse histological subtypes.^6^, ^7^, ^29^, ^30^, ^31^ Within the smaller subset of BL patients in our cohort (*n* = 13), we observed no instances of CTLS but four cases of LTLS. Indeed, the occurrence of LTLS is scarcely reported. A study conducted on a cohort of 102 lymphoma patients documented an occurrence of 42% for LTLS and 6% for CTLS. Within this investigation, nine cases of BL were observed, with six instances of LTLS and one case of CTLS, which concurs with our own observation of frequent occurrences of LTLS among BL patients.^5^ Furthermore, the incidence of TLS within the subset of patients with DLBCL and HGBCL in our cohort aligns closely with an international dataset of DLBCL patients treated with DA‐EPOCH‐R or R‐CHOP.^27^ This is noteworthy, considering that our patient population exhibits a higher incidence of elevated IPI at 75%, compared to 40% in the comparative cohort. Additionally, the median age of our cohort is 70 years, with a notable range from 31 to 95 years, a factor prominently reflected in the suboptimal 2‐year OS of 33%. Caution is warranted in making comparisons, given that our study population does not precisely align with the characteristics of the cohorts in the aforementioned clinical trials, however, these findings suggest that employing fractionated rituximab as an initial debulking strategy resulted in a low occurrence of TLS, like the observations made in other cohorts consisting of younger patients with less advanced‐stage lymphoma.

The standardization of approaches for preventing TLS remains a challenge. A recent Cochrane review highlighted the effectiveness of urate‐lowering agents in normalizing uric acid levels, but their impact on renal failure and mortality remains uncertain.^34^ Within our study, 26% of patients received rasburicase and it was observed that the occurrence of TLS was higher in this subset. This was indicative of an exceptional risk for TLS development within this subset, as the administration of rasburicase was not mandatory but based on the individual patient TLS risk score in our study. This underscores that urate‐lowering agents are just one component of the comprehensive TLS management strategy. Notably, tumor‐debulking approaches are concurrently employed. A prephase regimen administered before the initial cycle of immunochemotherapy, commonly utilizing steroids alone or in combination with vincristine or cyclophosphamide, is widely employed in acute lymphoblastic leukemia and high‐risk B‐cell lymphoma.^7^, ^29^, ^35^, ^36^, ^37^, ^38^, ^39^, ^40^, ^41^, ^42^, ^43^, ^44^ This strategy has demonstrated efficacy in reducing TLS and therapy‐related mortality.^32^, ^33^, ^36^, ^37^, ^38^, ^39^, ^40^, ^41^, ^42^, ^43^, ^44^, ^45^, ^46^ In our cohort, most patients (82%) received a steroid prephase, which may have contributed to the observed low occurrence of TLS following subsequent administration of fractionated rituximab. These results underscore the importance of implementing a comprehensive strategy for managing TLS in patients with high‐risk B‐cell lymphoma.

To the best of our knowledge, our real‐life study represents an innovative investigation into the potential of fractionated rituximab as a preemptive strategy for reducing the occurrence of TLS. Our findings indicate that implementing a comprehensive approach involving urate‐lowering agents, a steroid prephase, and fractionated rituximab tailored to individual patient risk profiles could result in a minimal occurrence of CTLS in high‐risk B‐cell lymphoma patients. Nevertheless, it is crucial to acknowledge the limitations inherent in the retrospective nature of this study and the small sample size. Additionally, potential confounding factors, particularly related to prephase use, should be considered when interpreting the results. Our results underscore the importance of further investigations, emphasizing the necessity for large‐scale prospective studies to validate and extend these observations.

## AUTHOR CONTRIBUTIONS

*Conceptualization*: Noémie Lang and Jerome Tamburini. *Methodology*: Noémie Lang and Jerome Tamburini. *Validation*: Noémie Lang, Jerome Tamburini, Kaveh Samii, Nicolas Mach, Martine Chamuleau, and Frank Stenner. *Formal analysis*: Noémie Lang and Jerome Tamburini. *Investigation*: Jasmine Mohamad and Noémie Lang. *Writing–original draft preparation*: Jasmine Mohamad and Noémie Lang. *Review and editing*: Noémie Lang, Jerome Tamburini, Thomas A. McKee, Antonin Bouroumeau, Kaveh Samii, Martine Chamuleau, and Frank Stenner. *Supervision*: Noémie Lang and Jerome Tamburini. All authors have approved the final draft of the manuscript.

## CONFLICT OF INTEREST STATEMENT

The authors have stated explicitly that there are no conflicts of interest in connection with this article.

## ETHICS STATEMENT

Ethical approval was obtained from the ethical committees of Geneva (CCER‐2020‐01659), Switzerland.

## Supporting information


**Table S1.** Therapeutic regimen of the cohort (*n* = 94).

**Table S2.** Tumor lysis syndrome preventive measures.

**Table S3.** Comparison of our real‐life cohort and available comparative datasets.

## Data Availability

All individual deidentified participant data collected during the trial will be shared following publication as per ICMJE statements, no end date.
