# Supplementary material for: Effectiveness of fractionated rituximab in preventing tumor lysis syndrome in aggressive B‐cell lymphoma: Insights from real‐life clinical practice
Source: Cancer Rep (Hoboken). 2024 Oct 16;7(10):e1983. doi: 10.1002/cnr2.1983 (PMC11480531; doi:10.1002/cnr2.1983)
Supplement: Supplementary file 1 — Table S1. Therapeutic regimen of the cohort (n = 94). [file CNR2-7-e1983-s003.docx]

**Table S1. Therapeutic regimen of the cohort (n=94)**

| Chemotherapy regimen, n (%) |  |
| --- | --- |
| R-CHOP-based | 54 (59) |
| DA-EPOCH-R | 8 (9) |
| R Hyper CVAD | 4 (4) |
| R-bendamustin | 12 (13) |
| Other R-chemo^†^ | 6 (61) |
| Rituximab single agent | 7 (7) |
| Missing | 1 (1) |

† Other regimen include : 5 R-CVP and one R-Gemcitabine

*Abbreviations: DA-EPOCH-R, dose adjusted: etoposide, prednisone, vincristine, cyclophosphamide, doxorubicin, rituximab; R-CHOP, rituximab, cyclophosphamide, doxorubicin, vincristine, prednisone; R-Hyper CVAD, rituximab, cyclophosphamide, doxorubicin, vincristine, dexamethasone, cytarabine, methotrexate*
