# Supplementary material for: Effectiveness of fractionated rituximab in preventing tumor lysis syndrome in aggressive B‐cell lymphoma: Insights from real‐life clinical practice
Source: Cancer Rep (Hoboken). 2024 Oct 16;7(10):e1983. doi: 10.1002/cnr2.1983 (PMC11480531; doi:10.1002/cnr2.1983)
Supplement: Supplementary file 2 — Table S2. Tumor lysis syndrome preventive measures. [file CNR2-7-e1983-s001.docx]

| Steroids prophase use, n (%) |  |
| --- | --- |
| Yes | 77 (82) |
| Missing | - |
| Allopurinol use, n (%) |  |
| Yes | 77 (84) |
| Missing | 3 (3) |
| Allopurinol dose (mg/day), mean (range) | 300 (50-300) |
| Rasburicase use, n (%) |  |
| Yes | 24 (26) |
| Missing | 2 (2) |
| Rasburicase dose (mg), mean (range) | 15 (7.5-22.5) |
| Rituximab dose (mg)* |  |
| Day 1, mean (range) | 224 (125-570) |
| Day 2, mean (range) | 224 (125-264) |
| Day 3, mean (range) | 224 (125-574) |

**Table S2. Tumor lysis syndrome preventive measures**

**Fractionated rituximab dose of 125 mg/m^2^ administered over 3 days (cumulative total of 375 mg/m²).*
