# Supplementary material for: Effectiveness of fractionated rituximab in preventing tumor lysis syndrome in aggressive B‐cell lymphoma: Insights from real‐life clinical practice
Source: Cancer Rep (Hoboken). 2024 Oct 16;7(10):e1983. doi: 10.1002/cnr2.1983 (PMC11480531; doi:10.1002/cnr2.1983)
Supplement: Supplementary file 3 — Table S3. Comparison of our real‐life cohort and available comparative datasets. [file CNR2-7-e1983-s002.docx]

**Tables S3. Comparison of our real-life cohort and available comparative datasets.**

**Table S3A. Burkitt lymphoma**

|  | Real-life BL cases (n=13) | Swiss BL dataset (n=20) |
| --- | --- | --- |
| Age, median (range) | 60 (45-75) | 70 (31-95) |
| Gender, n (%) |  |  |
| Male / Female | 8 (62) / 5 (38) | 48 (51) / 46 (49) |
| Stage, n (%) |  |  |
| Localized (I-II) / Advanced (II/IV) | 1 (8) / 12 (92) | 6 (6) / 88 (94) |
| ECOG, n (%) |  |  |
| 0-1 | 6 (46) | 15 (75) |
| 2-3 | 7 (54) | 5 (25) |
| Bone marrow involvement, n (%) |  |  |
| Present | 4 (31) | 5 (26) |
| Missing | 3 (23) | 1 (5) |
| Chemotherapy regimen, n (%) |  |  |
| R-CODOX / M-IVAC | - | 11 (55) |
| DA-EPOCH-R | 4 (31) | 9 (45) |
| R-CHOP | 2 (15) | - |
| R-Hyper-CVAD | 3 (23) | - |
| Other^†^ | 3 (23) | - |

† Other regimen include: one R-CVP regimen; one R-gemcitabine; one rituximab only as patient deceased shortly thereafter

*Abbreviations: BL, Burkitt lymphoma; DA-EPOCH-R, dose adjusted: etoposide, prednisone, vincristine, cyclophosphamide, doxorubicin, rituximab; DLBCL, diffuse large B cell lymphoma; ECOG, Eastern Cooperative Oncology Group; IPI, International Prognostic Index; R-CHOP, rituximab, cyclophosphamide, doxorubicin, vincristine, prednisone; R-CODOX / M-IVAC, rituximab, cyclophosphamide, vincristine, doxorubicin, methotrexate, ifosfamide, etoposide, cytarabine.*

|  | Real-life DLBCL/HGBCL cases (n=78) | CALGB 50303 dataset (n=473) |
| --- | --- | --- |
| Age, median (range) | 78 (31-95) | 57 (18-86) |
| Gender, n (%) |  |  |
| Male / Female | 38 (49) / 40 (51) | 263 (56) / 209 (44) |
| ECOG, n (%) |  |  |
| 0-1 | 51 (65) | 408 (87) |
| 2 | 13 (17) | 64 (14) |
| 3-4 | 9 (12) | - |
| Missing | 5 (6) | - |
| Stage, n (%) |  |  |
| Localized (I-II) / Advanced (III/IV) | 5 (6) / 73 (94) | 90 (20) / 371 (80) |
| IPI, n (%) |  |  |
| 0-2 | 18 (23) | 273 (60) |
| 3-5 | 58 (74) | 184 (40) |
| Missing | 2 (3) | - |
| Chemotherapy regimen, n (%) |  |  |
| R-CHOP-based | 49 (63) | 238 (50) |
| DA-EPOCH-R | 4 (5) | 235 (50) |
| R-bendamustin | 12 (15) | - |
| Other^†^ | 10 (13) | - |
| Missing | 1 (1) | - |
| Other^†^ | 3 (23) | - |

**Table S3A. High-grade B-cell lymphoma and Diffuse large B-cell lymphoma**

‡ Other regimen include: one hyper-CVAD, 3 R-CVP and 6 rituximab monotherapy

*Abbreviations: BL, Burkitt lymphoma; DA-EPOCH-R, dose adjusted: etoposide, prednisone, vincristine, cyclophosphamide, doxorubicin, rituximab; DLBCL, diffuse large B cell lymphoma; ECOG, Eastern Cooperative Oncology Group; IPI, International Prognostic Index; R-CHOP, rituximab, cyclophosphamide, doxorubicin, vincristine, prednisone.*
